# Minimal Stimulation Using Gonadotropin-Releasing Hormone Antagonist is Associated with Higher Live Birth Rates: A National Study of 13,050 Cycles

**DOI:** 10.1089/whr.2022.0080

**Published:** 2022-11-21

**Authors:** Emily G. Hurley, Fangbai Sun, Heping Zhang, Alex J. Polotsky, Julie Sroga Rios

**Affiliations:** ^1^ Department of Reproductive Endocrinology and Infertility, University of Cincinnati, West Chester, Ohio, USA.; ^2^ Department of Biostatistics, Yale University School of Public Health, New Haven, Connecticut, USA.; ^3^ Department of Obstetrics and Gynecology, University of Colorado Anschutz Medical Campus, Aurora, Colorado, USA.

**Keywords:** minimal stimulation, gonadotropin-releasing hormone antagonist, mini-IVF, mild IVF

## Abstract

**Background:**

The optimal protocol for minimal stimulation *in vitro* fertilization (IVF) has yet to be established. This study aims to determine if the use of gonadotropin-releasing hormone (GnRH) antagonist during minimal stimulation improves outcomes.

**Materials and Methods:**

All cycles designated as minimal stimulation from 2014 to 2016 from the Society for Assisted Reproductive Technology Clinic Online Reporting System were identified. Cycles in which GnRH antagonist was administered (*n* = 5984) were compared to those that did not receive it (*n* = 7066). Wilcoxon's rank-sum test and chi-square test were used to analyze continuous and categorical variables.

**Results:**

A total of 6750 patients undergoing 13,050 cycles were included. GnRH antagonist use was associated with a significantly higher total gonadotropin dosage (median 975.0 [interquartile range, IQR, 600.0, 1575.0] vs. median 660.0 [IQR 375.0, 975.0], *p* < 0.001), lower cycle cancelation rate (11.3% vs. 13.6%, *p* < 0.001; OR 1.24, 95% CI 1.12–1.38, *p* < 0.001), and higher live birth rate (4.3% vs. 2.1%, *p* < 0.001; OR 0.47, 95% CI 0.39–0.58, *p* < 0.001). GnRH antagonist use was associated with a significantly higher live birth rate in women ≥35 years of age (2.7% vs. 0.9%, *p* < 0.001; OR 0.34, 95% CI 0.25–0.47, *p* < 0.001) and antimullerian hormone <1 (4.9% vs. 2.6%, *p* = 0.004; OR 0.52, 95% CI 0.33–0.81, *p* = 0.004).

**Conclusion:**

The use of GnRH antagonist suppression during minimal stimulation IVF is associated with an improved live birth rate, especially in older women and in women with diminished ovarian reserve. Although GnRH antagonist use may increase costs, it significantly decreases cancelation rate, increases number of embryos cryopreserved, and should be encouraged for minimal stimulation IVF.

## Introduction

Minimal stimulation *in vitro* fertilization (IVF), also known as “mild” or “mini-IVF” is a type of protocol utilized in assisted reproductive technologies. Typically less medication is administered to stimulate a lower number of oocytes to develop compared to conventional IVF. Minimal stimulation may include the use of oral agents (clomiphene citrate, letrozole) and low-dose gonadotropins with and without the use of gonadotropin-releasing hormone (GnRH) antagonists. Nonetheless, a standardized definition and protocol regarding the recommended medications and dosing have yet to be established.

There are many advantages of minimal stimulation when compared to conventional IVF, including decreased exposure to medications, cost reduction, improved safety profile with decreased risk of hyperstimulation, and increased patient satisfaction with decreased stress.^1–5^ Cost savings have been estimated to be ∼40% when compared to conventional protocols.^6^ It is also estimated that 70% less medications are required.^7^ Minimal stimulation IVF is frequently used in patient populations with diminished ovarian reserve and also in women/couples with limited financial resources and/or in women with a good prognosis, for example, those with a history of tubal ligation.^8^ Previous studies have found an association between lower doses of stimulation medications and a limited number of oocytes retrieved with possible decreased risk of perinatal complications, including low birth weight.^3^

There is conflicting evidence regarding the outcome of live birth rate when comparing minimal stimulation to conventional IVF. Some studies have found similar pregnancy rates, others have found a lower ongoing pregnancy rate with minimal stimulation.^3,5,6,9^ However, there is good supporting evidence that in women considered to be poor responders, clinical pregnancy rates have been found to be similar.^7,8^ Nonetheless, the criteria of a “poor responder” is not well established.

Besides concern regarding decreased pregnancy rates, other disadvantages to minimal stimulation include possibly fewer oocytes retrieved, fewer embryos created, and thus fewer embryos cryopreserved.^6–10^ Also, patients may be at an increased risk of cancellation or having no embryos for transfer.^3,4,7,10^ Previously, studies have reported cancellation rates to be ∼18%–19%.^4^ GnRH antagonists are frequently included in conventional IVF cycles to prevent a premature surge of luteinizing hormone and thus ovulation.^6^ Using GnRH antagonists in minimal stimulation cycles may decrease the risk of premature ovulation resulting in a decrease in cancellation rate.

The purpose of this study was to evaluate whether the use of GnRH antagonist improves patient outcomes in minimal stimulation IVF. We hypothesize that the utilization of GnRH antagonists during minimal stimulation will not only decrease the cycle cancellation rate but also, more importantly, improve the live birth rate.

## Materials and Methods

Data were collected and verified by the Society for Assisted Reproductive Technology (SART) and reported to the Centers for Disease Control and Prevention in compliance with the Fertility Clinic Success Rate and Certification Act of 1992 (Public Law 102-493). The data in the SART Clinic Online Reporting System (CORS) were validated annually with some clinics having on-site visits for chart review based on an algorithm for clinic selection. During each visit, data reported by the clinic were compared with information recorded in patients' charts. Ten out of 11 data fields selected for validation were found to have discrepancy rates of ≤5%.^11^

This study was declared exempt by the Institutional Review Board at the University of Cincinnati (Study ID: 2018:71-36) and was approved by the SART Research Committee. All IVF cycles reported by SART CORS from 2014 to 2016 that were defined as “Minimal Stimulation” under “Patient Medication” for “ART Treatment” were collected. Any cycle with use of an “Agonist Flare” or “Agonist Suppression” used as patient medication was excluded. Demographic characteristics were collected, including female age, antimullerian hormone (AMH) level, body mass index, follicle stimulating hormone (FSH) level, smoking status, gestational history, and diagnosis.

The primary outcome studied was live birth per cycle. Secondary outcomes included cancellation rate, number of oocytes retrieved, number of embryos transferred, and number of embryos cryopreserved. Cycles marked as “Cycle Cancelled” in SART CORS were reported; however, the cause of cancellation was not further evaluated.

All cycles were analyzed as single cycles. The data were summarized as the number (percentages) or median (interquartile range [IQR]) as appropriate. Wilcoxon's rank-sum test was used for testing the difference between the two groups for continuous variables, and chi-square test was used for categorical variables. Univariate analysis was completed to evaluate variables associated with antagonist use. Multivariate logistic regression was used to establish a final model for antagonist use. Variables that were significant in the univariable analysis were introduced into the multivariable logistic regression analysis in a stepwise manner, using a *p*-value of <0.10 to enter and a *p*-value of <0.05 to remain. A generalized estimating equations logistic regression analysis was utilized to ensure that the results were unchanged among repeated measures from the same patient. Statistical significance was defined as a two-sided *p*-value <0.05. Analyses were performed with SAS, version 9.4 (SAS Institute).

## Results

### Patient demographics

A total of 6750 patients undergoing 13,050 cycles were included in the study, with 5984 in the antagonist suppression group and 7066 without antagonist suppression. The participants' age ranged from 19 to 54 years, median 41 (IQR 37, 44). The AMH level of participants ranged from undetectable (0) to 40 ng/mL, median 0 (IQR 0.0, 0.2). Women of younger age and with normal ovarian reserve were more likely to have received antagonist suppression (Tables 1 and 2). Those treated with letrozole were also more likely to have received antagonist suppression (33.9% vs. 21.2%, *p* < 0.001), while those treated with clomiphene were less likely (52.8% vs. 70.3%, *p* < 0.001) (Tables 1 and 2). Given the large number of cycles included in this study, many of the demographic categories were deemed statistically significant, however, these findings were likely not clinically significant.

**Table 1.** Patient Demographics and Cycle Characteristics in Minimal Stimulation Cycles with Antagonist Suppression or Without Antagonist Suppression

	With antagonist suppression (n = 5984)	Without antagonist suppression (n = 7066)	*p*
Patient age at start (year)	40.0 (36.0, 43.0)	41.0 (38.0, 44.0)	<0.001
AMH last value (ng/mL)	0.0 (0.0, 0.5)	0.0 (0.0, 0.0)	<0.001
BMI (kg/m^2^)	24.0 (21.5, 28.2), *n* = 3270	23.4 (21.0, 27.3), *n* = 2373	<0.001
Maximum FSH (IU/mL)	10.0 (7.0, 15.0), *n* = 3939	13.0 (9.0, 21.0), *n* = 5311	<0.001
Day of cancellation	9.0 (7.0, 12.0), *n* = 674	10.0 (6.0, 13.0), *n* = 964	0.045
Total 2PN	2.0 (1.0, 4.0), *n* = 4893	1.0 (0.0, 2.0), *n* = 5359	<0.001
Smoker	2270/5984 (37.9)	1487/7066 (21.0)	<0.001
Nulliparous	2568/5984 (42.9)	3019/7066 (42.7)	0.828
Male infertility	1178/5984 (19.7)	859/7066 (12.2)	<0.001
Endometriosis	337/5984 (5.6)	292/7066 (4.1)	<0.001
Polycystic ovaries	110/5984 (1.8)	116/7066 (1.6)	<0.001
Diminished ovarian reserve	4078/5984 (68.1)	5562/7066 (78.7)	<0.001
Tubal	784/5984 (13.1)	722/7066 (10.2)	<0.001
Uterine	314/5984 (5.3)	357/7066 (5.1)	<0.001
Unexplained	430/5984 (7.2)	368/7066 (5.2)	<0.001
Other non-infertile	808/5984 (13.50)	623/7066 (8.8)	<0.001
Clomiphene	3157/5984 (52.8)	4970/7066 (70.3)	<0.001
Aromatase inhibitors	2030/5984 (33.9)	1497/7066 (21.2)	<0.001
Dosage of FSH	975.0 (600.0, 1575.0), *n* = 5287	660.0 (375.0, 975.0), *n* = 3627	<0.001
Days of ovarian stimulation	12.0 (10.0, 13.0), *n* = 5309	11.0 (9.0, 13.0), *n* = 6084	<0.001

Data are presented as the number (%) or median (interquartile range).

2PN, 2 pronuclear; AMH, antimullerian hormone; BMI, body mass index; FSH, follicle-stimulating hormone.

**Table 2.** Patient Characteristics Associated with the Odds of Antagonist Suppression

Variables	Unadjusted analysis		Adjusted analysis	
	OR of antagonist suppression (95% CI)	*p*	OR of antagonist suppression (95% CI)	*p*
Patient age at start (year)	0.925 (0.919–0.932)	<0.001		
AMH last value (ng/mL)	1.236 (1.195–1.280)	<0.001		
BMI (kg/m^2^)	1.025 (1.015–1.036)	<0.001		
Maximum FSH (IU/mL)	0.968 (0.964–0.972)	<0.001		
Day of cancellation	0.976 (0.959–0.993)	0.006		
Total 2PN	1.173 (1.154–1.194)	<0.001		
Smoker				
Yes	Reference		Reference	
No	0.423 (0.391–0.457)	<0.001	0.437 (0.277–0.691)	0.010
Unknown	1.252 (0.964–1.627)	<0.001	1.063 (0.324–3.490)	0.420
Nulliparous				
Yes	Reference			
No	1.008 (0.940–1.080)	0.828		
Male infertility				
Yes	Reference		Reference	
No	0.565 (0.513–0.621)	<0.001	0.464 (0.270–0.799)	0.006
Endometriosis				
Yes	Reference			
No	0.722 (0.615–0.848)	<0.001		
Polycystic ovaries				
Yes	Reference			
No	0.891 (0.685–1.159)	0.390		
Diminished ovarian reserve				
Yes	Reference			
No	1.728 (1.598–1.870)	<0.001		
Tubal				
Yes	Reference			
No	0.763 (0.685–0.851)	<0.001		
Uterine				
Yes	Reference			
No	0.961 (0.822–1.123)	0.615		
Unexplained				
Yes	Reference			
No	0.710 (0.615–0.819)	<0.001		
Other non-infertile				
Yes	Reference			
No	0.928 (0.725–1.188)	0.553		
Clomiphene				
Yes	Reference		Reference	
No	2.123 (1.976–2.282)	<0.001	4.935 (2.963–8.219)	<0.001
Aromatase inhibitors				
Yes	Reference		Reference	
No	0.524 (0.484–0.566)	<0.001	0.516 (0.271–0.984)	0.044
Dosage of FSH	1.001 (1.001–1.001)	<0.001	1.001 (1.001–1.002)	<0.001
Days of ovarian stimulation	1.019 (1.011–1.027)	<0.001	0.893 (0.840–0.950)	0.0003
Cycle canceled				
Yes	Reference			
No	1.245 (1.121–1.383)	<0.001		
No. of oocytes retrieved	1.132 (1.118–1.145)	<0.001		
Embryo transfer attempted	1.300 (1.125–1.502)	<0.001		
Embryos cryopreserved	1.150 (1.122–1.178)	<0.001		
Clinical pregnancy				
Yes	Reference			
No	0.454 (0.378–0.544)	<0.001		
Live birth				
Yes	Reference			
No	0.475 (0.387–0.583)	<0.001		

Variables of nulliparous, polycystic ovaries, uterine, and other non-infertile were not introduced to the multivariate model since their univariate analysis *p*-values were not significant.

### Primary outcome

**FIG. 1.**
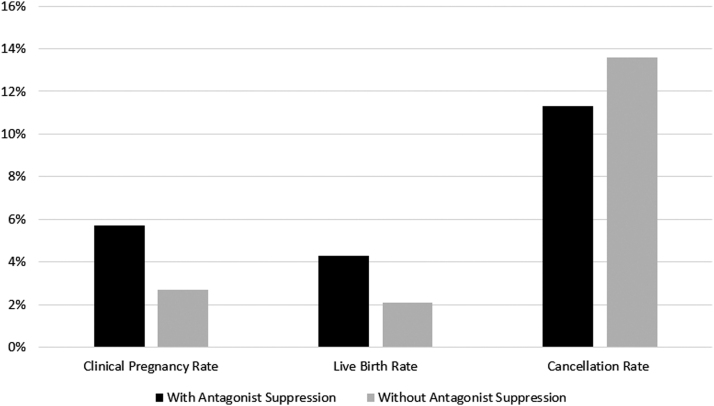
Gonadotropin-releasing hormone antagonist use improves outcomes in minimal stimulation in vitro fertilization.

The use of antagonist suppression was associated with a higher live birth rate (4.3% vs. 2.1%) (Fig. 1 and Table 3). When evaluating age range and AMH level specifically, improvement of live birth was significant in women with an AMH <1 and in women 35 years of age or older (Table 3). The live birth rate in women ≥35 to ≤39 years of age was 5.5% with antagonist versus 2.8% without antagonist (*p* < 0.001), and in women over the 40 years of age and older, the live birth rate was 1.1% with antagonist and 0.3% without (*p* < 0.001).

**Table 3.** Cycle Outcomes of Minimal Stimulation Cycles With and Without Antagonist Suppression

	With antagonist suppression (n = 5984)	Without antagonist suppression (n = 7066)	*p*
Cycle canceled	674/5984 (11.3%)	964/7066 (13.6%)	<0.001
No. of oocytes retrieved	3.0 (2.0, 6.0), *n* = 5310	2.0 (1.0, 3.0), *n* = 6102	<0.001
Number embryo(s) transferred	2.0 (1.0, 2.0), *n* = 1090	1.0 (1.0, 2.0), *n* = 605	<0.001
Embryos cryopreserved	1.0 (0.0, 2.0), *n* = 4943	0.0 (0.0, 1.0), *n* = 5411	<0.001
Clinical pregnancy	340/5984 (5.7%)	188/7066 (2.7%)	<0.001
Live Birth			
Overall	258/5984 (4.3%)	148/7066 (2.1%)	<0.001
0 < AMH <1	84/1712 (4.9%)	26/994 (2.6%)	0.004
AMH ≥1	97/907 (10.7%)	56/472 (11.9%)	0.512
Age <35	124/1005 (12.3%)	88/712 (12.4%)	0.989
Age ≥35	134/4979 (2.7%)	60/6354 (0.9%)	<0.001

Data are presented as the number (%) or median (interquartile range).

### Secondary outcomes

The use of antagonist suppression was associated with a higher total dose of FSH, lower cycle cancelation rate, greater number of oocytes retrieved, and higher number of embryos cryopreserved (Fig. 1 and Tables 1 and 3).

## Discussion

In this study, we evaluated whether or not the use of GnRH antagonist suppression provides benefit to minimal stimulation IVF. Our findings suggest that minimal stimulation cycles that utilize antagonist suppression result in improved patient outcomes, especially in women of advanced maternal age and/or those with a lower ovarian reserve. The benefits of antagonist suppression include a lower cancellation rate, increased number of oocytes retrieved, increased number of embryos cryopreserved, and improved clinical pregnancy and live birth rates.

There is a paucity of previous literature evaluating the use of GnRH antagonists with minimal stimulation IVF. A previous study published in 2002 compared 10 patients undergoing a minimal stimulation protocol with GnRH antagonists versus 10 patients undergoing minimal stimulation IVF without antagonists. The pregnancy rates (6/8 vs. 3/10) and implantation rates (9/20 vs. 4/24) were higher in the antagonist group; however, this was not found to be statistically significant.^6^ This article is supportive of our study results, however, the numbers included in the previous study were small and not powered to detect the difference. In addition, previous literature has found a decreased cancelation rate with GnRH antagonist use, which is supported by our findings.^9^

As mentioned in the Introduction, previous research has shown conflicting results when comparing the pregnancy rates of minimal stimulation with that of conventional IVF. Although this study did not compare minimal stimulation to conventional IVF directly, the live birth rate with minimal stimulation appears to be much lower than the nationally reported pregnancy rates for IVF based on maternal age alone, reported by the SART.^12^

The strengths of this study include the large population size provided by SART CORS. This is the largest study to date, that we are aware of, that assesses the use of antagonist suppression with minimal stimulation. Through our analysis, we were able to detect significant differences within the subgroup analysis of women ≥35 and AMH <1. This study, however, is not without limitations. One major limitation of the study is the lack of a universal accepted definition of minimal stimulation IVF. According to the “International glossary on infertility and fertility care,” mild ovarian stimulation for IVF is defined as “A protocol in which the ovaries are stimulated with gonadotropins, and/or other pharmacological compounds, with the intention of limiting the number of oocytes following stimulation for IVF.”^13^

However, per the “Data Dictionary” in the SART Documentation Portal, the definition of minimal stimulation states “Clinics may define minimal stimulation themselves.”^14^ Given the vague understanding of minimal stimulation, the number of cycles reported as minimal stimulation in SART is likely inaccurate. To improve our ability to study and optimize minimal stimulation IVF, a standard acceptable definition should be developed.

This study reports multiple significant differences in demographics between the two groups. However, given the large study size, including over 13,000 cycles, this can be explained by the statistical analysis of such large numbers. We do not believe that this reports clinical significance, as overall, the two populations are fairly similar. Potential confounding factors among the groups include the proportion with diminished ovarian reserve and the gonadotropin doses.

Some of the cycles included in the “without antagonist suppression” may have had another means of providing some degree of suppression. Previous literature has found that using clomid in addition to gonadotropin until day of trigger may assume the role of the GnRH antagonist by suppressing the luteinizing hormone surge while also decreasing cycle cost.^1,3^ Because duration of clomid is not able to be assessed through SART CORS, these cycles were not able to be extracted and looked at separately. The prolonged use of clomid may also have had a detrimental effect on the endometrium, with possible recommendation for cryopreservation of all embryos or have resulted in a decreased pregnancy rate, which cannot be accounted for in our study.^15^

Another limitation to this study was the exclusion of frozen embryo transfers. Nonetheless, we did find that there is a decreased chance of having an embryo(s) to cryopreserve when antagonist suppression is not used.

## Conclusion

Antagonist suppression should be recommended for all minimal stimulation IVF cycles. The use of antagonist suppression results in improved clinical outcomes, most importantly live birth rate. The increase in live birth rate is statistically significant in women of older age and those with a lower AMH. Antagonist suppression use may require an increase dose of gonadotropins and prolonged stimulation with increased ultrasound visits and thus result in an increase in overall costs. Moving forward, a cost-analysis should be performed to understand the impact of antagonist use on cycle costs and patients should be counseled accordingly. We also hope that this study encourages development of a standardized definition of minimal stimulation IVF with improvement of future research studies and creation of evidence-based protocols.

## Abbreviations Used

2PN: 2 pronuclear
AMH: antimullerian hormone
BMI: body mass index
FSH: follicle-stimulating hormone
GnRH: gonadotropin-releasing hormone
IQR: interquartile range
IVF: *in vitro* fertilization
SART: Society for Assisted Reproductive Technology
